# The Effects of Disinfectants on Dimensional Accuracy and Surface Quality of Impression Materials and Gypsum Casts

**DOI:** 10.4021/jocmr2009.04.1235

**Published:** 2009-06-21

**Authors:** Wala M. Amin, Muna H. Al-Ali, Sandra K. Al Tarawneh, Sahar Th. Taha, Mohamed W. Saleh, Nadia Ereifij

**Affiliations:** a Department of Prosthetic Dentistry, Faculty of Dentistry, University of Jordan, P.O. Box 13455, Amman 11942, Jordan; b Faculty of Dentistry, University of Melbourne, Australia; c Faculty of Dentistry, University of North Carolina, USA; d Faculty of Dentistry, University of Michigan, USA; e Research fellow and Clinical Dental Practitioner, Private practice, Amman, Jordan; f Department of Prosthetic Dentistry, Faculty of Dentistry, University of Jordan, Jordan

## Abstract

**Background:**

The study aimed to evaluating the effect of disinfecting impression materials on the dimensional accuracy and surface quality of the resulting casts.

**Methods:**

Impressions of a steel die constructed according to ANSI/ADA specification No.18 were made with each of alginate, addition cured silicone, condensation cured silicone and zinc oxide eugenol paste, and disinfected consequently by each of 0.2% chlorhexidine gluconate, 1% sodium hypochlorite, 2% gluteraldehyde for 5 minutes, and 0.5% sodium hypochlorite for 10 minutes. Dimensions of the disinfected impressions and their resultant casts were measured using a computerized digital caliper, and the dimensional changes were calculated. Reproduction of detail and surface quality of the resultant casts were assessed by grading casts surfaces according to a specific scoring system.

**Results:**

The 0.5% sodium hypochlorite was found to produce the least dimensional changes in all the impression materials. Corsodyl produced the maximum changes in both alginate and zinc-oxide eugenol while addition-cured silicon was most affected by Gluteraldehyde and condensation-cured silicon was most affected by Hexana. The dimensional changes, however, were minimal and clinically insignificant. Addition-cured silicon showed the best surface quality and dimensional stability followed by condensation-cured silicon. Alginate and zinc-oxide eugenol had poorer surface quality and were affected to a higher extent by the disinfection procedures.

**Conclusions:**

The results were comparable with the standard specifications for dimensional stability. Recommendations were made for the use of 10 minutes immersion in 0.5% sodium hypochlorite as the most appropriate disinfection protocol to the investigated impression materials.

**Keywords:**

Disinfectants; Gypsum casts; Impressions; Alginate; Addition-cured silicone; Sodium hypochlorite

## Introduction

Contamination of dental impressions with saliva and blood from the oral cavity occurs readily in dental clinics. Direct interaction between dental clinics and dental laboratories makes contaminated dental impressions difficult items to deal with from the cross infection point of view. Previous reports indicated that contaminated impressions can cross-infect gypsum casts that were poured against them [1]. Until 1991, rinsing impressions under running water was the recommended practice [2]. Guidelines for infection control in dental health care suggested that all dental prostheses and prosthodontic items should be cleaned, disinfected, and rinsed before they are handled in the laboratory using an active hospital disinfectant [3, 4].

Many studies have evaluated the effect of various disinfectants and methods of disinfecting impression materials, but the results of those studies varied widely. The role of a disinfectant should, ideally, be of a dual purpose, it must be an effective antimicrobial agent, yet cause no adverse response to the dimensional accuracy and surface features of the impression material and the resultant gypsum cast [5].

The dimensional stability of disinfected impressions had been a subject of investigation by many researchers who used a variety of approaches. Some studies used full arch casts [6-8] while others studied the effects on a die [8]. The measuring technique used in determining dimensional changes after disinfection also varied from using a Boley gauge [6], to the use of measuring microscope [9].

Therefore, the aim of the present investigation was to examine the effect of several disinfecting solutions on four commonly used impression materials. The objectives to achieve this aim were: (1) To assess the dimensional accuracy and surface quality of the impressions and the resultant gypsum casts using a purpose-made computerized digital caliper of high sensitivity (accurate to 0.001 mm); (2) To compare the results with the standard specifications; (3) To recommend, accordingly, a disinfection protocol for the corresponding impression materials.

The null hypothesis tested was that the different types of disinfectants used would produce similar effects on the dimensional accuracy and surface quality of the various impression materials evaluated and the resultant casts.

## Materials and Methods

The impression materials and disinfectants employed in this study were chosen to represent the most commonly used by dentists in Jordan (Tables 1 and 2).

**Table 1:** Impression materials and their recommended setting times

Impression material	Brand name/Supplier	Batch Number	Recommended setting time
Alginate	Alginoplast® fast set/Heraeus Kulzer/Holland	2557591	2 min
Add. Silicone	Elite H-D^+^/Zhermack Badia Polesine (Rovigo) Italy	C203035	8 min
Cond. Silicone	Bonasil light DMP LTD, E.U.	82350305 34930604	8 min
Zinc Oxide–Eugenol	SS White Group/England	120603	5 min

**Table 2:** The employed disinfectants and their recommended immersion times.

Disinfectant	Brand name/Supplier	Conc.	Batch Number	Recommended immersion time
Chlorhexidine Gluconate	Hexana/ M.H. Sider Health Care Prep./Jordan	0.2%	H 0142	5 min
Chlorhexidine Gluconate	Corsodyl/Glaxo Smith Kline/UK	0.2%	335E	5 min
Sodium Hypochlorite	Hypex/Jordan Chemical Industries Co. Ltd/Jordan	1.0%		5 min
Sodium Hypochlorite	Hypex/Jordan Chemical Industries Co.Ltd/Jordan	0.5%		10 min
Gluteraldehyde	Eimaldehyde solution Al-Eiman for cosmetics/Jordan	2.2%	05090947	5 min

**Figure 1:**
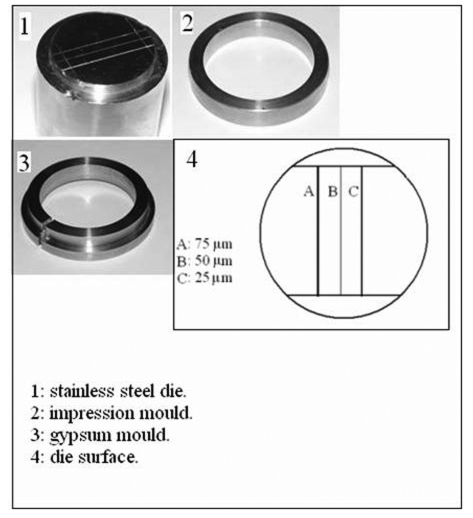
The parts "1, 2, 3" of the assembly constructed according to the ADA specification number 18 for testing dimensional stability and reproduction of details of impression materials. The diagram "4" representing the surface of the die showing the three vertical lines.

A stainless steel test die, an impression mould, a riser, and a gypsum mould were constructed according to ANSI/ADA specification No.18 [10] and BSI 12:1999 [11]. These are shown in Figure 1.

Impression materials were mixed according to manufacturers' instructions, the impression mould was placed on the die and a freshly mixed homogenous impression material was placed in the center of the die and spread to fill the mould. A flat plastic plate was placed over the mould and pressed the impression material firmly against the die assuring a positive metal to metal contact between the mould and the die. A metal flat weight of 1 Kg simulating the operator's finger pressure on a tray was placed over the plastic plate and the whole assembly was transferred into a water bath which was kept at 35^o^C ± 1 simulating the mouth temperature [12].

After the impression material had set, the assembly was removed from the water bath and the impression with the mould separated from the die. The test die and the mould were cleaned, dried and made ready for the next impression. After separation of the impression/mould assembly from the die, a riser was used to push the impression up to the same level of the surface of the mould.

Each impression surface was scanned prior to its disinfection using a two-dimensional computer scanner (BenQ scanner 2005) at a resolution of 500 Pixels/inch. The impression was then immersed in the disinfecting solution for the recommended time followed by rinsing in tap water at room temperature for 10 s. Twenty five impressions were made with each impression material, five impressions of each material for each of the five disinfectants. Additional five impressions of each material were immersed in sterile water for five minutes and used as control. Each impression was dried after rinsing with water and scanned in the same manner.

All impressions were poured within 1h after disinfection (the alginate impressions were kept in a sealed bag at 100% humidity) using dental stone (Elite ® Dental Stone / Zhermack, type IV framework dental stone) which was first wetted manually and then mixed on a vibrator for 30 s. Two hours after pouring the impressions, they were separated, and the resultant casts were coded and scanned at a resolution of 500 Pixel/inch. 

### Dimensional Stability

Measurements were made by one operator using a computerized digital calipers (C.D.C) tool that measured the distance between any two given points on a scanned image of an impression or a cast by counting the pixels between the two points on the scanned image of the impression/cast, and calculated the distance in millimeters [13]. Measurements were made between the cross lines, shown in Figure 1. Three readings were made for each stone cast: the first reading was for line "A", the second for line "B" and the third for line "C". The mean of the readings was taken for each cast and the dimensional change was calculated by using the following formula: (L - L'/ L) X 100, where L represents the dimensions of the die, and L' represents the mean dimension of the experimental cast (control or after disinfection). 

Data sets were treated statistically using analysis of variance (ANOVA) at 95% level of confidence and Fisher's Least Significant Difference for multiple comparisons.

### Surface quality

#### Detail reproduction of impressions

Impression were inspected visually without magnification and the accepted impressions were those that passed the ANSI/ADA specification for detail production which reproduced the full length of the 50μm-wide line for the alginate ,and the full length of the 25μm-wide line for the Zinc Oxide Eugenol and both the addition and condensation cured silicone impression materials.

#### Detail reproduction of stone casts

For assessment of detail reproduction of stone casts, the scanned images of the casts were inspected at X10 magnification and the following scoring system [12] with rating values from one to four was used as following: Rating 1: Well-defined, sharp detail and continuous line; Rating 2: Continuous line but with some loss of sharpness; Rating 3: Poor detail or loss of continuity of line; Rating 4: Marginally or completely not discernible line.

According to ANSI/ADA specification No.18 [10] dental stone casts made from alginate specimens have to reproduce the 75μm-wide line, and to satisfy specification No.19 [14] casts poured against silicone rubber must reproduce the 25μm-wide line.

For grading purposes both the 75μm and 50μm-wide lines were assessed for alginate [9] and only the 25μm-wide line was evaluated for Zinc Oxide Eugenol and the two silicone materials. Assessment of surface quality and evaluation of the detail reproduction of stone cast were carried out by one operator. The results were subjected to statistical analysed using Kruskal-Wallis test.

## Results

### Dimensional stability

**Figure 2:**
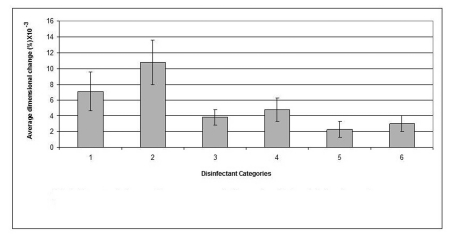
Dimensional changes for casts made from alginate impression material after disinfection.

**Figure 3:**
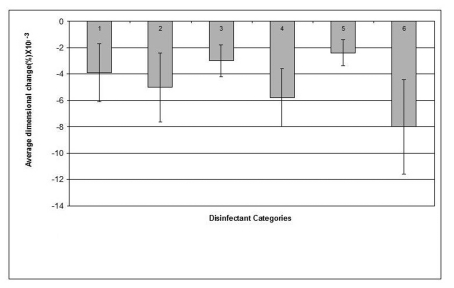
Dimensional changes for casts made from addition silicone impression material after disinfection.

**Figure 4:**
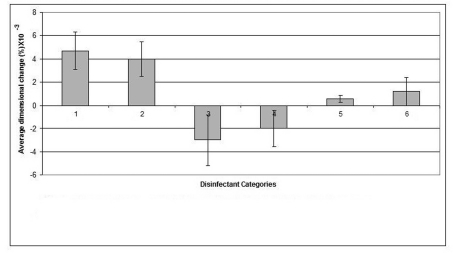
Dimensional changes for casts made from condensation silicone impression material after disinfection.

**Figure 5:**
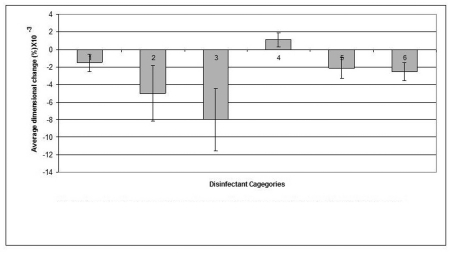
Dimensional changes for casts made from Zinc Oxide Eugenol impression materials after disinfection.

The mean value of the 15 measurements made on the casts, which resulted from pouring disinfected impressions, compared with corresponding measurements made on the steel die and controls are presented in Table 3. Dimensional changes of the casts made by pouring impressions of each material, namely, alginate, addition-cured silicone, condensation-cured silicone and zinc oxide-eugenol are presented graphically in Figures 2, 3, 4 and 5 respectively.

**Table 3:** The mean dimensional change for each impression material / disinfectant combination illustrating the dimensional change between the stone cast and the original die

Disinfectant	Alginate (mm)×10^-3^	Addition Silicone (mm)×10^-3^	Condensation Silicone (mm)×10^-3^	Zinc Oxide Eugenol (mm)×10^-3^
Hexana	7.1	-3.9	4.7	-1.5
Corsodyl	10.8	-5	4	-5
Sterile water	3.8	-3	-3	-8
1% Na-Hypochlorite	4.8	-5.8	-2	1.1
0.5% Na-Hypochlorite	2.3	-2.4	0.6	-2.1
Gluteraldehyde	3	-8	1.2	-2.5

The disinfectant that showed the least detrimental effect on the alginate impression material was 0.5% sodium hypochlorite which demonstrated as little dimensional change as 0.0023% and resulted in a cast with significantly improved dimensions (p < 0.05) compared to that of the water control (the cast which resulted by pouring an alginate impression after 10 min immersion in distilled water). "Corsodyl", on the other hand, was the disinfectant that caused the largest dimensional changes in alginate and incurred 0.108% swelling of the alginate impression.

Similar to its effect on alginate, sodium hypochlorite 0.5% disinfectant caused the smallest dimensional change (0.0024%) in the addition-cured silicone impression material and showed some improvement, although not significant, in the dimensions of the resultant casts compared to that of the water control. On the other hand, Gluteraldehyde caused the biggest change (0.008%) in the material's dimensions.

The condensation-cured silicone impression material demonstrated the least dimensional change (0.0006%) when disinfected by 0.5% sodium hypochlorite that showed a significant improvement (p < 0.05) in cast dimensions over the cast of the water control, whereas, Hexana disinfectant caused the largest dimensional change (0.0012%) in this impression material.

The zinc oxide-eugenol impression material showed a minimum dimensional change (0.0011%) following disinfection by 1% sodium hypochlorite and a maximum change in dimension (0.005%) after disinfection by "Corsodyl".

### Surface quality

**Figure 6:**
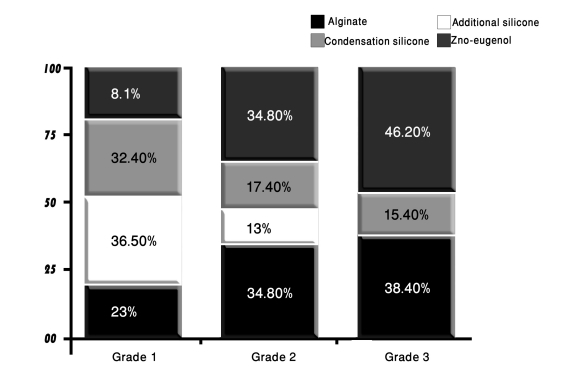
The distribution of impression materials according to their surface quality grades scored after disinfection.

The results of evaluating the effect of the disinfectants on the surface integrity of the stone casts revealed that 70% of the casts were grade 1; 19.2% grade 2 and 10.8% demonstrated grade 3 surface quality. None of the examined casts showed grade 4 surface. These results are illustrated in Figure 6.

The majority of the casts that showed grade 1 surface were those made from pouring addition-cured silicone impression material (36.5%), followed by those resulted from pouring condensation-cured silicone impressions (32.4%); and those resulted from pouring alginate impressions (23%), whereas only 8.1% of grade 1 surface quality impressions were demonstrated by casts made from pouring disinfected zinc oxide-eugenol impressions.

Grade 2 quality surfaces were mostly of casts that resulted from pouring disinfected alginate and zinc oxide-eugenol impressions (34.8% each) and partly of casts resulted from pouring condensation-cured silicone impressions (17.4%) and addition-cured silicone impressions (13%).

The casts that showed grade 3 surface quality were mostly those poured from zinc oxide-eugenol impressions (46.2%) followed by those made by pouring alginate impressions (38.4%). The condensation-cured silicone impressions produced casts that formed 15.4% of grade 3 surface quality casts; whereas none of the casts that resulted from pouring the addition-cured silicone impression were rated as grade 3 quality surfaces.

## Discussion

### Dimensional changes of the stone casts

None of the disinfectants had a discernable adverse effect on the accuracy of the resultant casts. In fact, some disinfectants such as 0.5% sodium hypochlorite significantly improved the dimensions of the resultant casts. This has confirmed the findings of some of the previous studies [5, 9, 15], while it contrasted with other studies that demonstrated adverse effects of elastomer immersion in disinfecting solutions [16].

Stone casts resulted from pouring decontaminated alginate impressions using the employed disinfectants showed slight dimensional shrinkage. This may be attributed to the processes of syneresis and imbibitions to which alginate was subjected. Alginate impressions were kept in a humid atmosphere for 1h prior to pouring the stone casts, during which the material might have been subjected to syneresis which caused shrinkage of the impression, but upon pouring the impressions with stone, the alginate imbibed the moisture of the stone mix which caused swelling of the impression and partly compensated for the syneresis shrinkage. Of all the disinfectants employed for decontaminating alginate impressions, sodium hypochlorite 0.5% affected the least changes in the dimensions of the resultant casts. This disinfectant, in fact, improved cast dimensions compared to those of the water control. These findings were consistent with the previous studies which reported that no significant distortion of alginate impressions could result from decontaminating these impressions by a variety of disinfectants [7, 17].

Stone casts that resulted by pouring disinfected addition-cured silicone impressions demonstrated a minimal reduction in dimensions caused by a marginal swelling of the impressions. Compared to the other impression materials investigated in this study, addition-cured silicone demonstrated superior dimensional stability. The changes in the dimensions of the resultant casts produced by pouring disinfected addition-cured silicone impressions were extremely minimal, clinically insignificant and within the range of changes that is acceptable by the standard specifications [14]. Once again, sodium hypochlorite 0.5% disinfectant affected some improvement in the dimensions of the casts that resulted by pouring addition-cured silicone impressions compared to the casts of the water control.

The stone casts poured from condensation-cured silicone impressions that were disinfected by each of: sodium hypochlorite 0.5%, gluteraldehyde, Corsodyl and Hexana demonstrated slight expansion as a result of shrinkage that took place in the condensation-cured silicone impression material upon disinfection. Whereas, casts produced by pouring the condensation-cured silicone impressions following disinfection by 1% sodium hypochlorite displayed a marginal reduction in dimensions as a result of swelling of the impression material upon exposure to 1% sodium hypochlorite. These differences may be attributed to the nature of the setting reaction of the condensation-cure silicone rather than to the disinfectant itself. This is consistent with previous studies [18-21] which pointed that care should be taken when working with condensation-cured silicone impression which may uncontrollably contract due to the loss of byproducts. Similar to the other investigated impression materials, the condensation-cured silicone produced stone casts that exhibited minimal dimensional changes when the impressions were disinfected by 0.5% sodium hypochlorite. These casts were closer in dimensions to the steel die than were the water control casts.

Casts produced by pouring disinfected zinc oxide-eugenol impressions demonstrated slight reduction in dimensions caused by marginal swelling of zinc oxide-eugenol impression upon exposure to the disinfectants except to 1% sodium hypochlorite which affected a minimal and clinically insignificant expansion of the resultant casts.

To this end, the observed differences in the behavior of the impression materials when exposed to disinfectants which affected some changes in the dimensions of the resultant casts may be attributed partly to the different characteristics of the impression materials themselves, and partly to the different disinfection regimens used.

**Figure 7:**
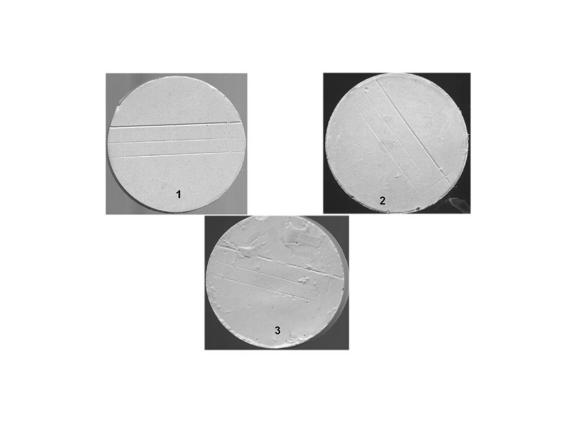
Surface quality and detail reproduction (X10 magnification): 1- sharp detail, continuous line; 2- continuous lines but with some loss of sharpness; 3- rough appearance with loss of continuity of the line.

### Surface quality

The effect of disinfectants on the surface quality of impressions indicated that the silicones, both addition and condensation-cured types, were superior in terms of surface integrity to the other impression materials. This is due to the hydrophobic nature of silicones which made the surface of these impressions highly resistant to the attack by the aqueous disinfectants regardless of their types or the length of exposure period. On the other hand, alginate impression material imbibed the disinfectants which, in turn, inflicted a discernable damage to the alginate impression surface.

Similar surface effects were displayed by zinc oxide-eugenol impressions which in spite of the oily nature of its surface, the tested disinfectants seemed to have attacked the impression surface chemically and inflicted a noticeable damage. The extent of this damage was proportionally related to the concentration of the disinfectant and the length of exposure of the impression to the disinfectant.

### Summary

In the light of the findings of the present investigation, the following conclusions can be obtained: (1) The five disinfectants employed in the present study affected all four impression materials very marginally and the stone casts obtained from disinfected impressions showed minimal dimensional changes that can be considered clinically insignificant. (2) Silicone impression materials of the two types, addition-cured and condensation-cured types, displayed superior qualities in terms of dimensional stability and surface integrity over alginate and zinc oxide-eugenol impression materials. The latter two materials, although showed minimal changes in dimensions, but were less stable compared to the silicones. (3) Stone casts obtained from alginate and zinc oxide-eugenol impression materials were of comparable dimensional stability and surface quality following each of the disinfection procedures. (4) Decontaminated addition-cured silicone impressions using all the employed disinfectants produced stone casts with dimensions very closely comparable to those of the standard metal die. (5) Of all the disinfectants employed in the present investigation, 0.5% sodium hypochlorite affected the least changes in the dimensions of the four impression materials and had a negligible effect on the quality of the materials' surface. The use of 0.5% sodium hypochlorite can, therefore, be recommended for disinfection by 10-minutes immersion of alginate; zinc oxide-eugenol; addition-cured silicone and condensation-cured silicone impressions prior to transporting these impressions to the laboratory.
